# Virtual and augmented reality in dental education: The good, the bad and the better

**DOI:** 10.1111/eje.12871

**Published:** 2022-11-20

**Authors:** Nikita Dzyuba, Jai Jandu, Julian Yates, Evgeny Kushnerev

**Affiliations:** ^1^ Division of Dentistry University of Manchester Manchester UK; ^2^ Department of Oral and Maxillofacial Surgery University of Manchester Manchester UK; ^3^ Academic Clinical Lecturer, Department of Oral and Maxillofacial Surgery University of Manchester Manchester UK

**Keywords:** augmented reality, dental education, higher education, pandemic education, virtual reality

## Abstract

**Introduction:**

Virtual reality and augmented reality (VR/AR) are becoming established technologies with a wide range of possibilities in various academic fields, including dentistry. The practice of dentistry encompasses a spectrum of skills and knowledge of anatomy, complex technical and clinical skills and sound academic understanding. This review aims to scope the current use of these technologies in dental education, explore their impact on teaching and learning and envisage their potential in this field.

**Materials and Methods:**

The Cochrane Library, PubMed and EMBASE were searched. Cochrane Handbook was used to conduct this systematic review. Inclusion and exclusion criteria were applied; randomised control trials published in English in the last 10 years (2010–2020) were considered and screened independently by two authors.

**Results:**

Fourteen of 524 studies were included and assessed. The majority of articles describing the use of VR/AR focused on an Undergraduate/General Dental Practitioner audience. Its use in Oral and Maxillofacial Surgery, Endodontics and Restorative dentistry was also described. There is evidence of motor skill acquisition using these systems which is comparative to that of traditional methods.

**Conclusion:**

The use of VR/AR is well established in dental education; most applications relate to undergraduate education as a useful adjunct to dental training. In this article, the breadth of learning in dental education using VR/AR was exploited providing an overview to aid dental education. VR/AR is a useful adjunct to conventional learning in dentistry. However, there are limitations preventing VR/AR widespread use and applications, such as lack of trials, standardisation and accreditation of systems/content.

## 1 INTRODUCTION

### 1.1 Background

The concept of VR originates from the 1830s when the first stereoscope was invented and used to project an image using a set of mirrors, giving the user a feeling of depth and immersion.^1^ VR‐like haptic features were later introduced in 1929 via a flight simulator used to train US air force pilots; this system was able to mimic air turbulence and plane movements.^1^ In the early 1960s, Ivan Sutherland first introduced and set into motion “virtual reality” as we now know it, by creating the first head‐mounted display.^2^ In the 1970s, VR development was chiefly driven by NASA and military training needs through the creation of various vehicle simulators.^3^ By the late 1980s and early 1990s, VR systems had become coupled with haptic gloves and computer systems, including part of NASA's astronaut training projects, and latterly were even used to help drive the Mars rovers.^1^ Publicly available VR systems also gained huge popularity as video games, in the form of arcade machines, such us Virtuality, Sega VR and Virtual Boy by Nintendo.^1^ Since 2012, new VR systems, incorporating headsets, were introduced to the public and commercially released: Oculus, Project Morpheus and HTC VIVE. These systems ignited a new wave of interest towards virtual reality and augmented reality (VR/AR) and use now extends outside of the gaming sphere into social networking, skills training and education, to name a few. VR is now used in many spheres, ranging from training pilots to military simulations and education. AR is also making its way into everyday life through mobile applications. There are now numerous uses of VR/AR technology, and examples of common applications can be seen in Table 1.

**TABLE 1:** Common applications of different VR/AR types

VR	AR
Oculus Rift HTC Vive Samsung Gear VR Xbox 360 Kinect CAVE PlayStation VR Google Cardboard 3D VR Microsoft HoloLens	Pokémon Go Snapchat filters GPS guidance applications Sky Guide AR Google Goggles IKEA Place BMW i Visualiser

### 1.2 Definitions

**FIGURE 1:**
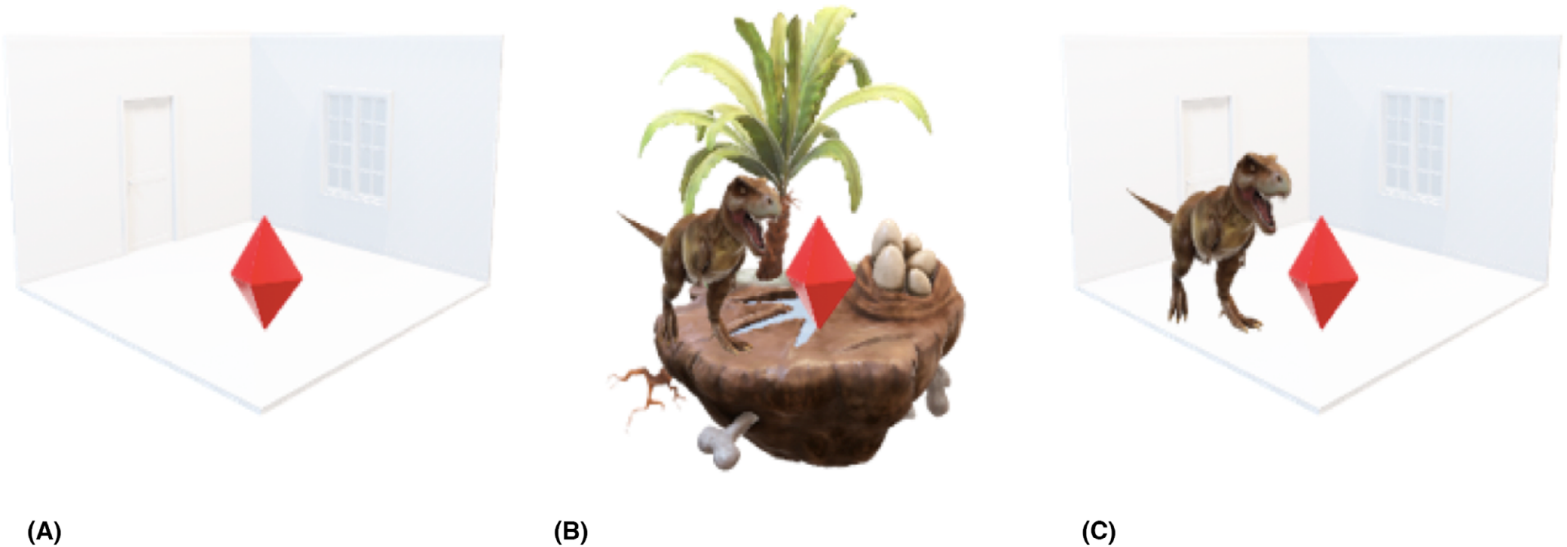
The red diamond represents an observer, physically in the same space, throughout. (A) Reality; the observer in a room, (B) virtual reality; Reality is replaced with a computer‐generated environment (either via headset or screen) and (C) augmented reality; a computer‐generated object is superimposed, enhancing reality (instant feedback on performance). A key point being in both virtual and augmented reality that the observer can interact with the computer‐generated objects.

As VR/AR are emerging technologies definitions vary widely; the Oxford English Dictionary defines VR as “a computer‐generated simulation of a lifelike environment that can be interacted with in a seemingly real or physical way…”^4^ and AR as “the addition of computer‐generated output, such as images or sound, to a person's view or experience of his or her physical surroundings by means of any of various electronic devices”.^5^ Broadly, VR can be thought of as immersion into a computer‐generated environment. Contrastingly, AR can be thought of as superimposition of a computer‐generated environment on reality. Figure 1 presents the concepts of VR/AR.

Succinctly: VR replaces reality whilst AR supplements it.^6^

Generally, VR/AR technology systems are apparent as head‐mounted devices with OLED screens, either with or without haptic controllers, which allow the user to interact with virtual models/features of applications. Professionally orientated systems, such as the Virteasy dental trainer and Simodont dental trainer (VR system), often consist of 3D displays, which show a virtual tooth, jaw models and instruments, and usually include a “handpiece‐replica” device, which provides haptic feel. Alternative systems, such as the DentSim dental trainer (AR), have slightly different design characteristics. Those include absence of haptic feel but real computer‐tracked handpiece and real plastic tooth models capable of augmenting the reality through presenting a virtual 3D model of the real‐time preparation, giving instant feedback on‐screen.

### 1.3 Scope and aims

The practice of dentistry encompasses a unique spectrum of skills; the teaching of which incorporates in‐depth head and neck anatomy and physiology, complex technical skills (restorative, endodontic, periodontic, prosthodontic and surgical therapy), subtle clinical skills (from communication to clinical judgement), sound academic understanding and leadership skills, to name a few. The breadth of learning in dental education provides many areas where VR/AR may be exploited to aid teaching.

In this review, we have gathered recent evidence to highlight the current trends of VR/AR use in dental education and to scope the direction of developing VR/AR technologies in this field. Besides the common applications of VR/AR in the gaming, military and commercial fields, applications in medical and dental education are also fast evolving.^7^, ^8^, ^9^, ^10^ Recent developments in dental and medical education range from VR/AR systems that supplement general anatomy teaching, enhance the acquisition of restorative and operative skills, to those that allow the practice of complex oral, orthognathic and maxilla‐facial surgery.

The SARS‐CoV‐2 pandemic has raised questions as to VR/AR place in participating in the delivery of dental education in line with social distancing regulations.^11^, ^12^, ^13^ These systems can potentially be used by students and dental professionals to learn, maintain and develop skills, remote from clinical and teaching premises.

This article is aiming to scope the evidence for current applications of VR/AR in dental education and to anticipate the potential uses of these technologies in this field. Only the highest‐quality studies [randomised control trials (RCTs)] were assessed to provide an update on the most reliable evidence.

## 2 MATERIALS AND METHODS

### 2.1 Search strategy

An electronic database search of CENTRAL via The Cochrane Library, PubMed and EMBASE via OVID was carried out using the following search terms “dentistry”, “virtual reality”, “dental education”, “augmented reality”, “simulation” and “dentistry education”; see Appendix 1.

### 2.2 Inclusion and exclusion criteria

The inclusion criteria were studies published from 11/05/2010 to 15/11/2020, relating to dental education and VR and/or AR and of RCT design. Earlier published articles were included in recently published reviews, and although providing stimulating information does not contribute to overall evidence. Exclusion criteria were studies not published in English, inability to access the full text and articles that describe computerised/web‐based or non‐immersive virtual reality. Each study was screened by two authors independently for suitability, the CASP and CONSORT checklists were used to aid the assessment, any disagreements were resolved with a third author.^14^, ^15^

### 2.3 Selection and appraisal

**FIGURE 2:**
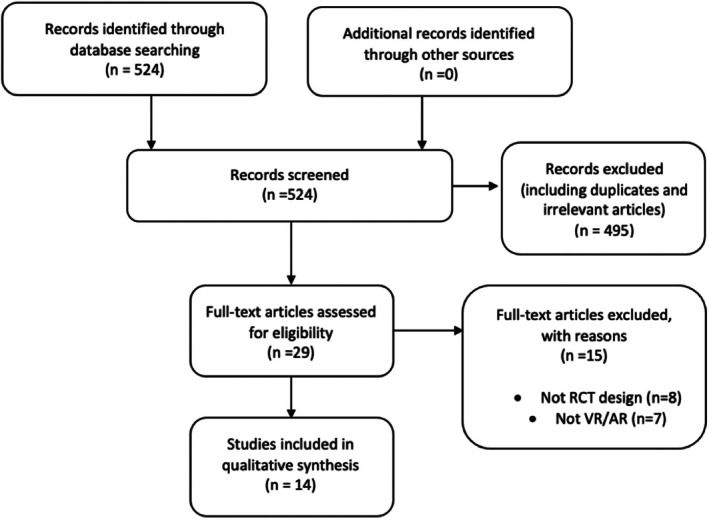
Flowchart of selected studies

Figure 2 highlights the selection process, see Appendix 2 for the list of articles excluded after initial screening. All included articles were read in full and scanned for current uses of VR and/or AR, in addition to any potential uses relating to dental education. All included articles were RCTs and so underwent quality assessment using the Cochrane Risk of Bias tool as a guide, see Appendix 3.^16^, ^17^

## 3 RESULTS

After electronic database screening, 524 articles were initially found. After irrelevant studies and duplicates were removed, 29 articles were included for full‐text scrutinisation. Full texts were assessed and analysed, 14 of 29 were included. Fifteen articles were excluded due to irrelevant study design and not being related to the main topic of the review (see Appendix 2). The earliest included article was published in December 2010 and the latest in August 2020.

### 3.1 Uses of VR/AR in dental education

Table 2 highlights the key data extracted from the included RCTs.

**TABLE 2:** Key data from RCTs

Study	Area	Population	Intervention	Outcome	Results
Mladenovic et al. (2020)^22^	Paediatrics	21 fourth‐ and fifth‐year dental students	Anaesthetic infiltration for anterior superior alveolar nerve in paediatric patients over six presented at the children's department. Intervention group—practised with AR‐supported device in a dental office 2 h weekly for a period of 2 weeks as well as traditional theoretical and plastic model training	Average time from needle cap removal to anaesthetic administration Salivary cortisol levels	Intervention group—faster anaesthesia administration (*p* < .05) There was no statistically significant difference between the groups in the level of cortisol in saliva
Murbay et al. (2020)^19^	Undergraduate	32 second‐year undergraduate dental students	Use of VR—Moog Simodont dental trainer haptic system for pre‐clinical training	Performance on carrying out a simple operative procedure (cavity preparation for direct restoration)	Higher percentage of satisfactorily performance in group with VR training (*p* < .05)
Vincent et al. (2020)^23^	Undergraduate	88 first‐year undergraduate dental students	Cavity preparations practice: Intervention group—VR haptic simulator—Virteasy HRV Simulation, Control group—conventional work on plastic analogue teeth Cavity preparation on plastic teeth for test	Drilling skills—evaluated by: percentage of tissue removed corresponding to the required target; drilling time and quality of cavity preparation	Improvement in both groups; similar results in performance of final test
de Boer et al. (2019)^29^	Undergraduate	126 first‐year dental students, without prior experience of cutting teeth	Use of VR—Moog Simodont dental trainer haptic system with different levels of force‐feedback, for simple operative procedure (cavity preparations) Investigate an ability to transfer operative skills at different levels of force feedback	Satisfactory completion of cavity preparation Questionnaire to assess the experience of working with different levels of force feedback	Skills learnt at one level of force‐feedback are transferable to others The questionnaire showed that most students noticed when they worked at a different level of force feedback
Mladenovic et al. (2019)^20^	Undergraduate	41 fourth‐ and fifth‐year undergraduate dental students	Practising administering inferior alveolar nerve block (IANB): Intervention group—practised with AR‐supported device 2 h per week for 4 weeks, plus theoretical teaching and demonstration on plastic models. Control group—theoretical teaching and demonstration on plastic models only	Measured average time for performing anaesthesia, heart rate whilst performing and anaesthetic success Post‐clinical questionnaire evaluating their knowledge and skills	Intervention group—faster average time (*p* < .05). Specifically, shorter time between uncapping of the needle to receiving a negative aspiration test in procedure for IANB. No difference in success (*p* = .219) No difference in heart rate (*p* = .293) Had a higher average score and/or a more limited range of responses on each item of the questionnaire
Dwisaptarini et al. (2018)^24^	Undergraduate	32 sixth year dental students. No previous experience of the simulator	Training for caries removal: Intervention group—practised on two micro‐CT multi‐layered carious teeth using the visuo‐tactile AR simulator Control group—practised on two extracted teeth On test day each participant performed a minimally invasive caries removal on one extracted tooth	Performance score (minimally invasive caries removal task) analysed using specially designed score scale Secondary outcome measures were tooth mass loss and task completion time	Improvement in performance score of both groups; no difference in between groups No differences in tooth mass removed and task completion time
Pulijala et al. (2018)^18^	OMFS	95 Indian OMFS residents from seven dental schools	Intervention group—VR surgery application on an Oculus Rift with Leap Motion device to interact with the anatomy, data, and instruments routinely used in the surgery through their VR experience Control group—similar content in a standard PowerPoint presentation on a laptop	Measured confidence levels using a Likert scale Objective assessment of cognitive skills (oral surgery knowledge)	Intervention group—increased self‐confidence (*p* = .034). Those in the first year of their training showed the greatest improvement Intervention group—an overall knowledge improvement pattern, however, no difference than control group (*p* = .025; *p* = .024)
de Boer et al. (2017)^30^	Undergraduate	101 first‐year dental students. “No previous experience in cutting a tooth or working in a VLE with force‐feedback were included in this study”	Use of VR—Moog Simodont dental trainer haptic system for cavity preparation. Group 1—practised without force‐feedback Group 2—practised with force‐feedback All practiced and tested under both circumstances, but in a different order	Performance score Completed a questionnaire to evaluate student satisfaction	All students who performed operative task in Group 1 failed the test. Group 2—performed significantly better (*p* = .031 and *p* = .008) 100% of the students preferred working with force‐feedback
Al‐Saud et al. (2017)^27^	Undergraduate	63 participants with no previous dental training, with a level of education to a typical undergraduate dentistry cohort	Use of VR—Moog Simodont dental trainer haptic system for cavity preparation shapes exercise Group 1—AR haptic dental simulation with online continuous feedback Group 2—verbal feedback from a qualified dental instructor Group 3—a combination of instructor and device feedback	Skill retention—immediately after training, at 1 week and at 1‐month Task performance measured by percentage of task completion (amount of target tissue removed), preparation time and error score in percentage	Group 3—substantially better performance and fewer errors (*p* = .006) Group 3—improved performance in skill retention and generalisation of knowledge to novel tasks (*p* < .001)
de Boer et al. (2016)^31^	Undergraduate	124 first‐year students, without previous experience in cutting a tooth or working in a virtual learning environment	45 min practice in what: Group 1—2D vision Group 2—3D vision Test using the vision they had practised in. After the first test all of the students switched the type of vision	Completed a questionnaire that was evaluating their preferences of 2D or 3D training experience Number of exercised completed within 45 min Mean preparation time	93% of participants preferred 3D vision Group 2—achieved significantly better results in operative task than students who worked in 2D (*p* = .031; *p* = .025) No differences in mean drilling time between groups
Kikuchi et al. (2013)^28^	Undergraduate	43 fifth‐year dental students	Use of VR—Moog Simodont dental trainer haptic system for practising porcelain fused to metal crown preparation Group 1—AR feedback with instructor feedback Group 2—AR feedback without the instructor's feedback Group 3—neither used feedback features of AR, nor instructor feedback All performed PFM crown preparation under the same setup once a week for 4 weeks	Total scores (damage to mesial adjacent tooth, damage to distal adjacent tooth, occlusal reduction, wall incline, retention, resistance, wall smoothness, margin location, chamfer width, inter‐proximal clearance, finish line continuity, undercut) Preparation time	Group 1 and 2—significantly higher total scores (*p* < .05) Between group 1 and 2 the instructor did not result in a significant difference for training, whilst it shortened the preparation time at early stages (*p* < .05)
Suebnukarn et al. (2012)^21^	Endodontics	10 junior endodontic postgraduate trainees with limited surgical experience (performed 1–2 endodontic microsurgical procedures)	Group 1—performed pre‐surgical practice using the VR haptic devices for first endodontic microsurgery. Then endodontic microsurgery with no virtual pre‐surgical practice Group 2—performed their first endodontic microsurgery without virtual pre‐surgical practice, followed by endodontic microsurgery with virtual pre‐surgical practice	Primary outcome—quality of performance assessed using the endodontic surgical competency rating scale	Higher performance scores when practised endodontic microsurgery on fresh cadaveric porcine after practising on presurgical VR simulation systems (*p* = .041) Significantly higher performance scores for molar tooth osteotomy noted when participants completed presurgical AR simulation practice (*p* = .042)
Suebnukarn et al. (2011)^25^	Undergraduate	32 fourth‐year dental students with no prior experience with the simulation	AR haptic simulator training using microcomputed tomography (micro‐CT) tooth models on minimising procedural errors in endodontic access preparation Group 1—training on the micro‐CT tooth models with a haptic AR simulator Group 2—training on extracted teeth using a phantom head Post training exercise: participants from both groups performed an access opening on extracted maxillary molar adjusted in phantom head	Main outcome—procedural errors Secondary outcome—tooth mass loss and task completion time	Improvement in error score in both groups (*p* < .05). No difference between groups. No difference in error score reduction between groups was noted Group 1—decreased the amount of hard tissue volume lost on the post‐training exercise (*p* < .05). No difference in task completion time. No difference in task completion time between both groups
Suebnukarn et al. (2010)^26^	Undergraduate	32 sixth‐year dental students…no prior experience with the simulation	AR haptic simulator training for performing endodontic access tooth preparation using different types of augmented kinematic feedback and practising bimanual dexterity using various dental instruments Group 1—received augmented kinematic feedback via force Group 2—received augmented kinematic feedback via mirror views Group 3—received augmented kinematic feedback via force and mirror views Group 4—control, no augmented kinematic feedback	Endodontic access preparation performance was scored by the: visibility of the canal orifices, each four axial walls, and the pulpal floor Skills acquisition and retention Mean task completion time	Groups 1, 2 and 3 (all that received augmented kinematic feedback) had a higher endodontic access preparation performance score at the earliest stages of training only (*p* < .05). No difference by Day 2 of the acquisition session

#### 3.1.1 VR use in dental education

Oral and maxillofacial surgery (OMFS) trainees reported increased confidence (*p* = .034) when using immersive VR (Oculus Rift with Leap Motion) surgery applications to practice compared to conventional methods alone.^18^ Knowledge amongst those who trained with the VR surgery application also outscored (*t* = 2.331; df = 50; *p* = .024) those who trained with conventional techniques that consisted of 2D videos and photographs.^18^

#### 3.1.2 AR use in dental education

For the teaching of technical operative skills, AR applications that out‐performed conventional methods were commonly described.^19^, ^20^, ^21^, ^22^ AR applications that equalled the performance of conventional methods, in technical skills teaching, were also common.^23^, ^24^, ^25^, ^26^ AR applications used adjunctively to conventional methods were also described to increase operative skills acquisition.^27^, ^28^ The use of haptic simulation coupled with AR further enhanced the realism of the experience, though this was not necessarily a requirement for the effective teaching of students.^23^, ^29^

The reviewed articles have been divided into groups depending on the type of simulation system used.

##### Moog Simodont dental trainer

The application of a haptic VR simulation (Moog Simodont dental trainer) in the practice of cavity preparations, resulted in greater satisfactory performance (*p* < .001) amongst undergraduate students.^19^ Force feedback and 3D vision were key components in enhancing performance (*p* < .001) and student approval, and de Boer et al.^29^ found there was transferability of acquired manual dexterity skills to different levels of force.^30^, ^31^ Additionally, the results obtained through conducting questionnaires amongst participants showed a significant level of preferability of using systems with forced feedback (*p* < .05).^30^ A combination of both traditional assessors and haptic VR simulation feedback is most beneficial for students acquisition and retention of basic dental skills; better student performance (*p* < .001) and fewer errors (*p* = .006) compared to simulation system feedback or conventional feedback alone.^27^

##### Virteasy dental trainer

Caries removal practice using an VR simulator (Virteasy dental trainer) resulted in drilling skills equal to that acquired by conventional methods (*p* < .01 for both control and study groups). Compared to the conventional group, there was also a reduction in the time taken to perform the procedure (*p* < .01; *p* < .001: 22% improvement from 543 ± 73 to 424 ± 105 s), less iatrogenic damage and less supervision and teaching time required throughout.^23^

##### SensAble OMNI and SensAble PHANToM OMNI haptic device

Haptic AR simulation (SensAble Omni haptic device), which can be connected to a standard personal computer system and monitor, showed to be as effective as training on extracted teeth to prepare students in visuotactile caries identification and its removal following minimally invasive techniques (*p* < .05).^24^ Pre‐surgical practice with this device connected to a laptop led to an increased quality of performance in endodontic postgraduate student's molar microsurgery and osteotomy procedures (*p* = .041; *p* = .042).^21^ Cavity preparation training with this device resulted in improved performance equal to conventional teaching methods regarding student performance (*p* < .05) and decreased the amount of hard tissue lost from the procedure (*p* < .05).^25^ The use of AR haptic simulation feedback (SensAble PHANToM OMNI haptic device) led to a reduction in the time taken to complete endodontic access opening amongst novice users (*p* < .05).^26^

##### DentSim dental trainer

AR simulator DentSim required increased time for students to become familiar with the systems in the absence of a trained instructor. Students that received feedback from either the instructor or the system performed better at operative skills than students that received no feedback (*p* < .05); however, the time taken to perform these skills was increased (*p* < .05).^28^

##### Mobile‐application‐based systems

An AR simulation (using a mobile device in a headset and specially designed patterned models) for Inferior Alveolar Nerve Block (IANB) training was described and resulted in students performing more successful and faster anaesthesia (*p* < .05). The post‐clinical questionnaire showed that students that used headsets rated their knowledge and skills significantly higher (*p* < .05).^20^

A similar study showed that training with an AR mobile application led to students to complete Anterior Superior Alveolar infiltrations faster in paediatric patients than those who used theoretical teaching and plastic models alone (*p* < .05). The authors also investigated the levels of cortisol in the saliva of the participants to evaluate student stress when anestheticising, however, found no statistically significant difference.^22^

### 3.2 The potential use of VR/AR in dental education

Table 3 highlights the potentials of VR/AR mentioned in the reviewed RCTs.

**TABLE 3:** Potentials of VR/AR mentioned in the reviewed RCTs

Author	Title	Potentials
Mladenovic et al. (2020)^22^	Effect of augmented reality simulation on administration of local anaesthesia in paediatric patients	AR applications may help students control and manipulate anaesthetic syringes when first anesthetising paediatric patients for dental treatment
Murbay et al. (2020)^19^	Evaluation of the introduction of a dental virtual simulator on the performance of undergraduate dental students in the preclinical operative dentistry course	As well as the assessment of current students, VR systems have the potential to help with admission tests and as a way to accredit external examinations for qualified dentists
Vincent et al. (2020)^23^	Contribution of Haptic Simulation to Analogic Training Environment in Restorative Dentistry	VR systems have a strong potential to be an efficient “pedagogical adjunct” for enhancing the learning process and practising fine motor operative skills. Additionally, VR systems can be considered further to be implied in microsurgery and robotic surgeries
De Boer et al. (2019)^29^	The Effect of Variations in Force Feedback in a Virtual Reality Environment on the Performance and Satisfaction of Dental Students	VR with forced feedback experience has high potential to be used for practising precision for advanced fine tasks and is highly useful in the dental curriculum.
Mladenovic et al. (2019)^20^	Effectiveness of Augmented Reality Mobile Simulator in Teaching Local Anaesthesia of IANB	AR has the potential to be used in oral and maxillofacial surgeries for identifying anatomical reference points Potential to be used more in dental morphology applications AR mobile simulators have the potential to be used effectively for learning local anaesthesia in preclinical and clinical settings
Dwisaptarini et al. (2018)^24^	Effectiveness of the Multilayered Caries Model and Visuo‐tactile Virtual Reality Simulator for Minimally Invasive Caries Removal: A Randomised Controlled Trial	VR and AR technologies have an excellent potential to be used in simulators that are focusing specifically on minimally invasive caries removal training
Pulijala et al. (2018)^18^	Effectiveness of Immersive Virtual Reality in Surgical Training—A Randomised Control Trial	The application of VR in oral surgery and maxillofacial facilities has great potential, as it allows to visualise anatomy in 3D and closed‐up perspective. The implication of VR in OMS training would be highly beneficial
De Boer et al. (2017)^30^	The Effect of Force Feedback in a Virtual Learning Environment on the Performance and Satisfaction of Dental Students	VR systems with haptic force feedback technology, such as Simodont, have the potential to be further implemented in dentistry training facilities for mastering manual dexterity skills
Al‐Saud et al. (2017)^27^	Feedback and motor skill acquisition using a haptic dental simulator	VR simulations offer the opportunity to be accepted in various surgical disciplines, as they provide continues feedback, both audio‐visual and tactile. Additionally, VR has good potential to be used for operative dentistry motor skills learning
De Boer et al. (2016)^31^	Student performance and appreciation using 3D versus 2D vision in a virtual learning environment	VR systems have the potential to be used effectively by undergraduate students for training in dental operative manual dexterity exercises
Kikuchi et al. (2013)^28^	Evaluation of a Virtual Reality Simulation System for Porcelain Fused to Metal Crown Preparation at Tokyo Medical and Dental University	VR simulation systems, such as DentSim, have a great potential to be used within dental clinical skills facilities for operative skills training
Suebnukarn et al. (2012)^21^	The use of cone‐beam computed tomography and virtual reality simulation for pre‐surgical practice in endodontic microsurgery	VR simulation systems have an excellent potential to be used in endodontic and endodontic microsurgery pre‐surgical training and general practising
Suebnukarn et al. (2011)^25^	Access cavity preparation training using haptic virtual reality and microcomputed tomography tooth models	VR simulation systems possess a great potential to be used as an endodontics access cavity preparation teaching and training tool for dental students
Suebnukarn et al. (2010)^26^	Augmented Kinematic Feedback from Haptic Virtual Reality for Dental Skill Acquisition	VR haptic systems show great potential and importance to be used further for operative, including endodontic, clinical skills training and as a reliable student assessment tool. Authors recommend incorporating haptic VR systems into dental skill training programs

### 3.3 Quality assessment summary

All, except Pulijala et al.^18^ study, had limited or ambiguous reporting of their randomisation processes. Similarly, this trend is seen regarding the reporting of allocation concealment, with only three studies presenting low risk.^21^, ^24^, ^25^ Due to the nature of the interventions relating to VR/AR use in education, the participants, in all studies, are likely to be aware of being in the experimental or control group, creating difficulties in achieving successful blinding. Most studies had blind assessment of the outcome/s or extracted data that were unlikely to be affected; however, some studies provided concerns or uncertainty regarding this.^31^ The majority of studies, excluding de Boer et al.^29^, ^30^ studies, had no missing outcome data or justified minor dropouts, and all the studies reported on all their outcomes of interest. All the studies, except for Dwisaptarini et al.^24^ presented additional risks of bias deemed to be significant by the authors.

**FIGURE 3:**
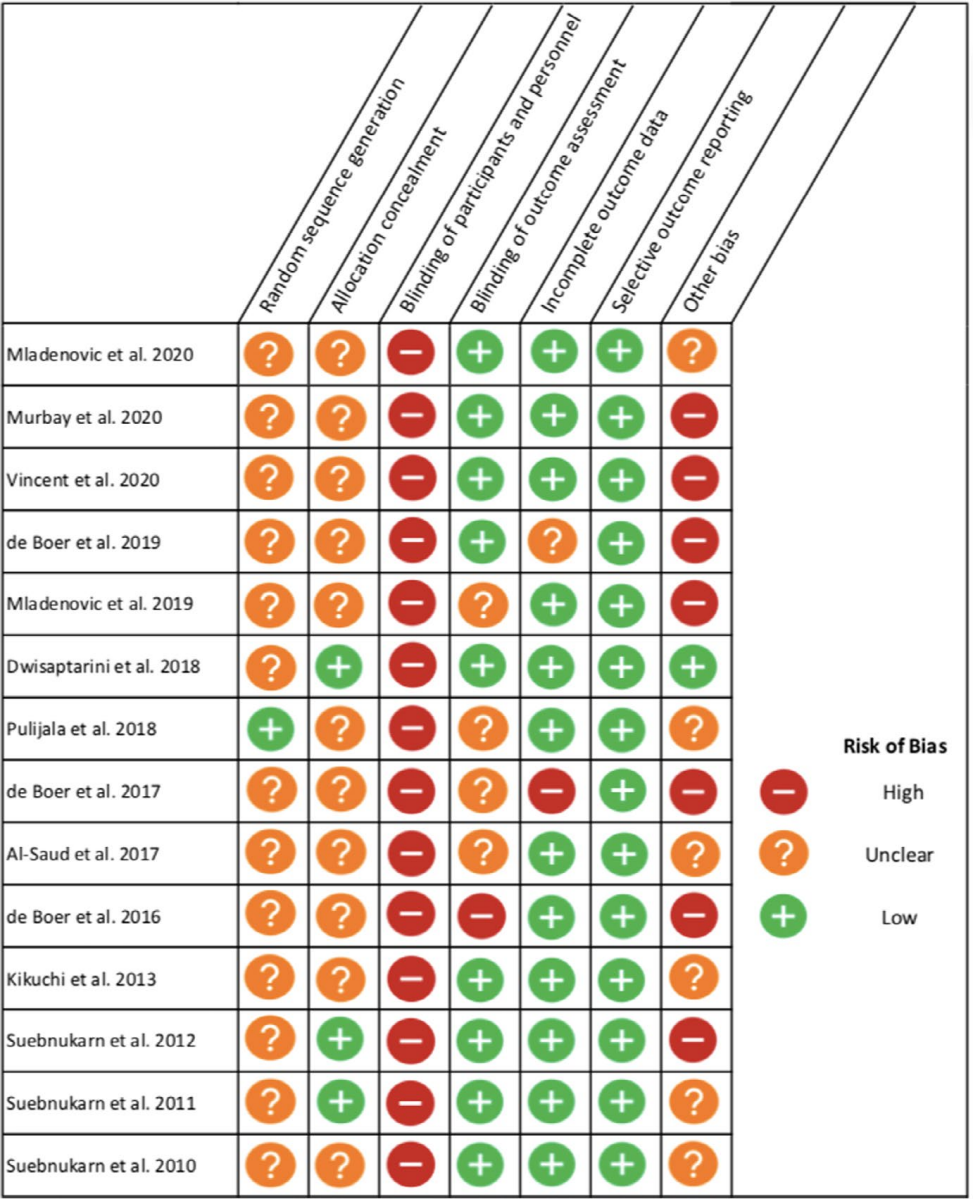
Risk of bias summary

Figure 3 summaries the internal validity of the included RCTs, see Appendix 3 for detailed assessment.

## 4 DISCUSSION

Murbay et al.^19^ study concluded that AR can be used to effectively teach direct restorations at the pre‐clinical stage and as an adjunct may allow faster transition from pre‐clinical to clinical study. The authors do not recommend it in isolation due to the lack of large sample studies across geographic areas and institutions.

Vincent et al.^23^ and Dwisaptarini et al.^24^ found no difference between conventional methods and AR training and recommended them as an educational option that could save staff supervision and teaching time, especially with further refinement.

Interestingly, Al‐Saud et al.^27^ who looked at the usefulness of AR system feedback with or without instructor feedback, found the former to be much superior to just AR feedback alone. This indicates that individual student use of AR systems is not equivalent to use in a clinical environment with tutors on‐hand.

However, they did find those with haptic feedback had a more cautious strategy and removed less material, which is particularly advantageous in the advocation of minimal invasive dentistry. The authors also highlight that haptic AR allows students to increase practice time, whilst not increasing tutor need, this may be especially suited for socially distanced set‐ups. Kikuchi et al.^28^ showed that an AR system better‐trained students in crown preparations than conventional methods when instructor feedback was absent. This suggests that AR systems with automatic feedback are more useful than conventional practice techniques when used by students alone.

Mladenovic et al.^20^ investigated mobile AR applications and found when used for training resulted in more effective first IANBs suggesting better knowledge and learning of the procedure. A similar system helped to train students better for administering anaesthetic infiltrations on children, however, did not find a difference in the student's stress levels.^22^

de Boer et al.^31^ showed the benefit of utilising 3D vision to improve the acquisition of manual dexterity skills. They also found it was greatly preferred by students. de Boer et al.^30^ found that for novice users force‐feedback is an essential component for AR training systems to be effective for the teaching of motor skills. The use of force‐feedback is also preferred by students. Significantly, de Boer et al.'s^29^ study exampled that AR simulators do not have to exactly replicate clinical reality for students to be able to acquire motor skills as there is a degree of transferability. However, it is uncertain if this translates outside of the “airotor” handpiece.

Suebnukarn et al.^21^ investigated the effectiveness of using VR haptic system to practise endodontic microsurgery. The authors concluded that the pre‐surgical use of the system clearly improved the endodontic surgery performance and success of the actual procedure and advocated implementation of AR pre‐surgical training to enhance the care quality and further improve patient safety standards.

Earlier, Suebnukarn et al.^25^ study identified that training on both conventional phantom heads and AR simulators can successfully reduce procedural errors during endodontic access. Participants who honed their skills using the AR system also showed a more conservative (minimally invasive) approach to reducing tooth tissue.

Suebnukarn et al.'s^26^ study showed that AR haptic simulation with augmented feedback can be effective in skills acquisition at the early stages of training; users evidenced better outcome scores as well as reduced tasks completion time.

Pulijala et al.'s^18^ study concluded that the use of VR for Oral Surgery training can significantly improve self‐confidence in addition to the knowledge levels of trainees. The effectiveness of the VR was found to be especially significant at the early stages of education; this suggests that it a useful tool for development of pre‐clinical to clinical skills.

### 4.1 Current trend

The usefulness of VR/AR systems in dental education is comparative to that of “updated” current training simulators (e.g. plastic teeth, anatomical models, phantom heads, actors, etc.). Simulation training provides valuable experience and is a long‐established method of teaching in dentistry; VR/AR systems offer a new exciting era of realism, enhancing the learning experience. The lack of high‐quality RCTs exploring the efficiency of VR is surprising; however, it may represent a shift towards AR technology, which most of the included articles evaluated.

There are extensive applications of VR for the undergraduate audience, AR applications are too mostly evidenced in undergraduate teaching and include ID block and operative training simulators. Cavity preparation and caries removal simulators are common, and soft tissue simulations to teach periodontal pocket probing, for example, have also been described.^32^ Additionally, some studies describe the use of virtual reality as a way to create a new teaching environment which assists with the learning of tooth anatomy and morphology.^33^

There are clear benefits compared to traditional teaching methods such as freedom from the time constraints of formal schedules and instant feedback, allowing the possibility for self‐paced learning.^23^, ^24^, ^34^ In addition, VR/AR systems may be used irrespective of access to clinical spaces, anatomy specimens and clinical teaching facilities.^35^, ^36^ There is the capability of these systems to provide additional aids, for example giving constant computerised feedback on the operator's actions, which provides an obvious benefit for students.^23^, ^37^ These systems produce less waste than conventional plastic models, constituting a more environmentally friendly form of practise.^23^, ^29^ Additionally, the fact that no aerosol is generated with most VR/AR dental training systems makes them a great option for dental training during public health crises, such as the SARS‐CoV‐2 pandemic. These systems are positively rated by students and from a faculty perspective, they require reduced supervision and instruction from tutors.^20^, ^23^, ^24^, ^27^, ^28^, ^38^, ^39^ This is particularly relevant at the current time; the SARS‐CoV‐2 pandemic has altered the landscape for dental education for at least the near future.^12^, ^13^ VR/AR systems may provide part of the solution; students would not require access to clinical spaces or assessors to be physically present and be able to develop/maintain their operative skills outside of university facilities. This is conditional on the quantity of devices available; it is imperative to maintain best‐practice hygiene standards if sharing any devices between students.

Generally, there is uncertainty regarding these systems which are preventing their widespread implementation. These include a limited precedent for the cost–benefit ratio of these systems, high purchase and potential maintenance costs and a lack of standards for software.^18^, ^21^, ^24^, ^28^, ^40^ There are technological challenges such as lack of visual alignment with the operative field (e.g. several non‐immersive AR systems project an image on a separate monitor), the inability of AR to account for patient movements correctly and “ghosting”, a double image which may occur with certain types of 3D vision techniques.^28^, ^31^, ^40^ The discomfort of using VR headsets for extended periods and motion sickness experienced by some users is not to be forgotten.^36^ Though there are clear benefits of these technologies, they have not been scrutinised by decades of use; therefore, it is unwise for these systems to be used as a replacement to traditional methods. This may prevent the drive to implement such systems from a purely financial sense. Even though VR/AR use that is supplemental to traditional methods may increase the benefit gained from such systems, a combination of VR/AR systems and current teaching may provide the optimal educational benefit.^27^

VR/AR application mainly focuses on the education of technical skills; however, motor skills are only part of the skill set of competent dentists; application of these systems into holistic patient care education could be a potentially useful area of expansion.^19^ For example, VR scenarios for the identification of common medical emergencies in the dental setting or to prastice dealing with tricky patient situations. Virtual surgical environments may be used to educate inexperienced clinicians on the layout and etiquette of theatre.^18^ This has the potential to allow trainees to maximise their time in theatre by spending less time acclimatising to a new environment. VR may be applied to assessment methods, with the added benefit of objective evaluation, through relevant metrics.^19^, ^40^ An example is Objective Structured Clinical Examinations, where novel scenarios can be used to test student's reactions without the need for actors.^19^, ^40^ In the same regard, these systems can be used in the ever‐increasing demands of admission processes to help to discriminate between applicants.^19^, ^41^

The amalgamation of operative and situational skills is an exciting prospect; VR scenarios that allow the incorporation of patients' anamnesis, detailed surrounding circumstances and even patient mindsets, into the practice of technical skills.^19^ Yamaguchi et al.^42^ developed a patient face model that showed great potential for trainees to account for patients' reactions such as painful and worried looks in VR environments. Artificial Intelligence can be employed to simulate lifelike conversation, providing an even more “realistic” simulation of real clinical practice, similar to the ideas presented by Marei et al.^43^ and Marei et al.^44^ Learning of endodontic morphology requires the visualisation of complex internal structures of teeth, we see a VR model of the internal apparatus of teeth as a potentially useful tool in teaching endodontic anatomy. Dentists are required to be ongoing learners and to undergo CPD training after dental school; accreditation of VR/AR system content would allow this training to be supplemented with these systems.^19^, ^23^ Most current systems primarily target undergraduate‐level dental education, the transferability of adjunctive VR/AR into CPD and postgraduate dental education is anticipated.

These systems have the potential to encourage active learning, whilst also supporting the migration away from passive teaching, e.g. traditional lectures. Their fundamental hands‐on nature and the capacity to incorporate scenarios and cases require users to engage in critical thinking, actively participate and take greater responsibility for their understanding.^45^

As VR/AR systems become more common, the use of these privately by students may provide a useful aid in the reading of clinical/technical degrees and even careers; as the internet liberated the gaining of knowledge, these systems have the potential to unshackle technical skills learning from the confines of university skills labs and expensive postgraduate courses.

This systematic literature review analyses and summarises articles that report on the use of VR/AR in dental education. A limitation of this review is that only articles relating to dental education were searched; there may have been influential articles outside of this field that could have been universally applied. The sample size and the quality of evidence are insufficient to provide reliable recommendations but do reveal the uses, advantages, disadvantages and potentials of these systems. Qualitative assessment with narrative synthesis to assess current trends was performed; quantitative synthesis was not undertaken due to the quality of included trials, heterogeneity of results and inconsistency in reporting. There is a need for further RCTs with a transparent and reproducible design. The authors recommend the use of CASP, CONSORT and the Cochrane Handbook as supporting tools in an effort to produce high‐quality evidence.

Overall, the results solidify that VR/AR systems have applicability in dental education and support that many systems are currently available. In undergraduate education, VR/AR systems have joined the cohort of simulation training methods (plastic teeth, anatomical models, phantom heads, actors etc.). In postgraduate education, these systems are used to educate clinicians in endodontic techniques. In OMFS, these systems have the potential to allow trainees to practise procedures more akin to real‐life surgeries with the benefit of additional feedback data. These systems also have use outside of surgical/technical applications, for example, training in medical emergency recognition and interactive academic courses. Key benefits include allowing self‐paced learning, providing instant feedback and the ability to provide additional aids such as feedback on posture. However, limitations such as technical issues (lag, high cost of maintenance, etc.), lack of standards (content, file formats, etc.) and issues regarding storage of confidential data are preventing widespread implementation.

## 5 CONCLUSION

This review has highlighted the potential of VR/AR to expand across the different aspects of undergraduate education, into CPD training, and to integrate technical training with clinical scenarios. This offers an exciting prospect for holistic teaching. Regarding formal dental education, a pragmatic approach would be to implement these systems alongside current ones, until long‐term studies advocate their use in isolation. Further RCTs and a meta‐analysis would be useful to evaluate the effectiveness of VR/AR teaching methods against tried and tested methods, before the implementation of these systems in isolation.

## CONFLICT OF INTEREST

The authors have no conflicts of interest to declare.

## Data Availability

The data that support the findings of this study are available in the results and appendices sections of this article.

## APPENDIX 1

### PUBMED SEARCH STRATEGY

((((((“dentistry”[MeSH Terms] OR “dentistry”[All Fields]) AND (“virtual reality”[MeSH Terms] OR (“virtual”[All Fields] AND “reality”[All Fields]) OR “virtual reality”[All Fields])) OR ((“dentistry”[MeSH Terms] OR “dentistry”[All Fields]) AND (“augmented reality”[MeSH Terms] OR (“augmented”[All Fields] AND “reality”[All Fields]) OR “augmented reality”[All Fields]))) OR ((“dentistry”[MeSH Terms] OR “dentistry”[All Fields]) AND (“Simulation”[Journal] OR “simulation”[All Fields]))) AND (“education, dental”[MeSH Terms] OR (“education”[All Fields] AND “dental”[All Fields]) OR “dental education”[All Fields] OR (“dentistry”[All Fields] AND “education”[All Fields]) OR “dentistry education”[All Fields])) AND “loattrfull text”[sb] AND “2010/05/11”[PDat]: “2020/11/15”[PDat] AND “humans”[MeSH Terms]) AND (“loattrfull text”[sb] AND “2010/05/11”[PDat]: “2020/05/07”[PDat] AND “humans”[MeSH Terms])

### EMBASE VIA OVID SEARCH STRATEGY

Database: Embase <1974 to 2020 November 15>.

Search Strategy:

1 ((([dentistry and virtual reality] or augmented reality or simulation) and dental education) or dentistry education).af. (516)

2 limit 1 to (human and yr = “2020”) (54).

3 limit 2 to randomised controlled trial.^4^

4 limit 3 to (english language and randomised controlled trial).^4^

### COCHRANE LIBRARY SEARCH STRATEGY

(virtual reality):ti,ab,kw OR (augmented reality):ti,ab,kw AND (dentistry):ti,ab,kw AND (education):ti,ab,kw OR (simulation):ti,ab,kw (Word variations have been searched)

Dental and Oral health domain. From 11/05/2010 to 15/11/2020

## APPENDIX 2

Full‐text articles excluded with reasons (*n* = 14) are presented in Table 4.

**TABLE 4:** Reasons for exclusion of articles read in‐full

Study	Title	Reason for exclusion
Zhan et al. (2020)	Evaluation of a dynamic navigation system for training students in dental implant placement	Not VR/AR
Liebermann & Erdelt (2020)^33^	Virtual education: Dental morphologies in a virtual teaching environment	Not RCT design
Mladenović et al. (2020)	The use of mobile‐aided learning in education of local anaesthesia for the IANB	Not related to VR/AR
Sameer et al. (2020)	Dynamically Navigated versus Freehand Access Cavity Preparation: A Comparative Study on Substance Loss Using Simulated Calcified Canals	Not related to VR/AR
Zafar, Lai, Sexton & Siddiqi (2020)^46^	Virtual Reality as a novel educational tool in pre‐clinical paediatric dentistry training: Students' perceptions	Not RCT design
Soltanimehr et al. (2019)^34^	Effect of virtual versus traditional education on theoretical knowledge and reporting skills of dental students in radiographic interpretation of bony lesions of the jaw	Not related to VR/AR
Marei et al. (2019)^44^	Collaborative use of virtual patients after a lecture enhances learning with minimal investment of cognitive load	Not related to VR/AR
Marei et al. (2018)^43^	The effectiveness of integration of virtual patients in a collaborative learning activity	Not related to VR/AR
Llena, Folguera, Forner, & Rodríguez‐Lozano, (2018)^47^	Implementation of augmented reality in operative dentistry learning	Not RCT design
Hayward et al. (2016)^48^	Script‐theory virtual case: A novel tool for education and research	Not RCT design
Wang, Zhao, Li, Zhang, & Wang (2015)^49^	Preliminary evaluation of a virtual reality dental simulation system on drilling operation	Not RCT design
Rhienmora et al. (2015)^40^	Recognising Clinical Styles in a Dental Surgery Simulator	Not RCT design
Allaire (2015)^50^	Assessing Critical Thinking Outcomes of Dental Hygiene Students Utilising Virtual Patient Simulation: A Mixed Methods Study	Not RCT design
Qi, Yan, Li, & Hu (2013)^51^	The Impact of Active Versus Passive Use of 3D Technology: A Study of Dental Students at Wuhan University, China	Not related to VR/AR
Gal, Weiss, Gafni & Ziv (2011)^52^	Preliminary assessment of faculty and student perception of a haptic virtual reality simulator for training dental manual dexterity	Not RCT design

## APPENDIX 3

Quality assessment of included studies is presented in Table 5.

**TABLE 5:** Quality assessment of included studies

Authors	Random sequence generation	Allocation concealment	Blinding of participants and personnel	Blinding of outcome assessment	Incomplete outcome data	Selective outcome reporting	Other bias
Mladenovic et al. (2020)^22^	Judgement: Unclear Comment: Insufficient information about the sequence generation process. “The participants were randomly divided into two groups (control and study groups)”	Judgement: Unclear Comment: No mention of how allocation occurred	Judgement: High Comment: Participants would likely be aware of using the intervention or not	Judgement: Low Comment: Data is unlikely to be affected by assessor's opinion	Judgement: Low Comment: No missing outcome data reported	Judgement: Low Comment: Report on all outcomes of interest	Judgement: Unclear Comment: No mention of funding source
Murbay et al. (2020)^19^	Judgement: Unclear Comment: Insufficient information about the sequence generation process. “undergraduate students were randomly selected”	Judgement: Unclear Comment: No mention of how allocation occurred. “undergraduate students were randomly selected”	Judgement: High Comment: Participants would likely be aware of using the intervention or not	Judgement: Low Comment: Data is unlikely to be affected by assessor's opinion; two independent assessment methods used, objective measurements	Judgement: Low Comment: No missing outcome data reported	Judgement: Low Comment: Report on all outcomes of interest	Judgement: High Comment: (1) The VR group were repeating the procedure unlike the control group, likely to be an advantage (2) “Relatively small sample size and inexperienced cohort group” (3) No mention of funding source or conflict of interest
Vincent et al. (2020)^23^	Judgement: Unclear Comment: Insufficient information about the sequence generation process. “All students were enrolled and randomly assigned”	Judgement: Unclear Comment: No mention of how allocation occurred. “All students were enrolled and randomly assigned”	Judgement: High Comment: Participants would likely be aware of using the intervention or not	Judgement: Low Comment: Data is unlikely to be affected by assessor's opinion; some of the results data was evaluated in a double‐blind manner	Judgement: Low Comment: No missing outcome data reported	Judgement: Low Comment: Report on all outcomes of interest	Judgement: High Comment: (1) The study “took place in only one academic dental institution, its results may not be generalizable to students in other programs” (2) “Study may have had human factor bias, and it used only one type of simulator and took place in only one area of dentistry” (3) No mention of funding source or conflict of interest
De Boer et al. (2019)^29^	Judgement: Unclear Comment: Insufficient information about the sequence generation process. “…randomly distributed into four groups”	Judgement: Unclear Comment: No mention of how allocation occurred. “…randomly distributed into four groups”	Judgement: High Comment: Participants would likely be aware of using the intervention or not	Judgement: Low Comment: Unclear if blinding occurred but data unlikely to be affected by assessor's opinion; set criteria used	Judgement: Unclear Comment: No missing outcome data for the primary outcome. Questionnaire only (*n* = 99) voluntary	Judgement: Low Comment: Report on all outcomes of interest	Judgement: High Comment: Authors affiliated institution, Academic Centre for Dentistry Amsterdam (ACTA), receives royalties per unit (Simodent dental trainer) as a return of investment in development
Mladenovic et al. (2019)^20^	Judgement: Unclear Comment: Insufficient information about the sequence generation process. “The participants were randomly divided into two groups: a control group with 19 students and an experimental group with 22 students”	Judgement: Unclear Comment: No mention of how allocation occurred. “The participants were randomly divided into two groups: a control group with 19 students and an experimental group with 22 students”	Judgement: High Comment: Participants would likely be aware of using the intervention or not	Judgement: Unclear Comment: Unclear if blinding occurred.	Judgement: Low Comment: No missing outcome data reported	Judgement: Low Comment: Report on all outcomes of interest	Judgement: High Comment: (1) An increase in heart rate itself may be an incomplete measure of stress (2) Subjective determination of success in anaesthesia (3) The study had a small number of participants and took place at only one dental school (4) No mention of any funding source
Dwisaptarini et al. (2018)^24^	Judgement: Unclear Comment: Insufficient information about the sequence generation process. “Participants were randomly assigned to either the experimental group (caries‐ removal training with micro‐CT multilayered caries model and visuo‐tactile VR simulator) or the control group (conventional training with carious extracted teeth)”	Judgement: Low Comment: “A statistician not involved with the study undertook the randomization using sealed opaque envelopes”	Judgement: High Comment: Participants would likely be aware of using the intervention or not	Judgement: Low Comment: “The main outcome measure in both groups was performance scores assessed by an expert blinded to trainee and training status”	Judgement: Low Comment: No missing outcome data reported	Judgement Low: Comment: Report on all outcomes of interest	Judgement: Low Comment: The study appears to be free of other sources of bias
Pulijala et al. (2018)^18^	Judgement: Low Comment: Researchers describe a random sequence generating software that was used. “A simple parallel randomization approach was followed in assigning the participants using a randomly generated number series on GraphPad Prism 7 software”	Judgement: Unclear Comment: No mention of how allocation occurred. “A simple parallel randomization approach was followed in assigning the participants using a randomly generated number series on GraphPad Prism 7 software”	Judgement: High Comment: Participants would likely be aware of using the intervention or not	Judgement: Unclear Comment: “The primary outcomes measure was the comparative evaluation scores of the perceived self‐confidence levels before and after the intervention, measured using a 5‐point Likert scale” Insufficient information regarding blinding of outcome assessment is available	Judgement: Low Comment: “4 residents from the control group withdrew from the study after answering the pre‐intervention questionnaire to attend emergency cases in the hospital” *n* = 4 dropped out of the control group (*n* = 40, at the start); total participants *n* = 91	Judgement: Low Comment: Report on all outcomes of interest.	Judgement: Unclear Comment: “Study had a small number of participants. Further research should involve larger sample size”
De Boer et al. (2017)^30^	Judgement: Unclear Comment: Insufficient information about the sequence generation process. “All students were randomly assigned to both groups by a lottery ticket”	Judgement: Unclear Comment: No mention of how allocation occurred.	Judgement: High Comment: Participants would likely be aware of using the intervention or not	Judgement: Unclear Comment: Unclear if blinding occurred but data unlikely to be affected by assessor's opinion; set criteria for the primary outcome. However, interpretation of secondary outcome may be affected by lack of blinding	Judgement: High Comment: Insufficient information on why data is missing “101 and 78 students participated in test 1 and test 2, respectively. From these, only the data of sixty‐two students who precisely adhered to the testing protocol were used for statistical analyses”	Judgement: Low Comment: Report on all outcomes of interest	Judgement: High Comment: Authors affiliated institution, Academic Centre for Dentistry Amsterdam (ACTA), receives royalties per unit (Simodent dental trainer) as a return of investment in development
Al‐Saud et al. (2017)^27^	Judgement: Unclear Comment: Insufficient information about the sequence generation process. “Participants were randomly allocated to one of three groups”	Judgement: Unclear Comment: No mention of how allocation occurred. “Participants were randomly allocated to one of three groups”	Judgement: High Comment: Participants would likely be aware of using the intervention or not	Judgement: Unclear Comment: Data unlikely to be affected by assessor's opinion: “Dental task performance was captured using the following metrics provided automatically by the simulator”	Judgement: Low Comment: No missing outcome data reported	Judgement: Low Comment: Report on all outcomes of interest	Judgement: Unclear Comment: No mention of funding source
De Boer et al. (2016)^31^	Judgement: Unclear Comment: Insufficient information about the sequence generation process. “One hundred and twenty‐four first‐year students were randomly divided into two groups”	Judgement: Unclear Comment: No mention of how allocation occurred	Judgement: High Comment: “The students were familiar with the type of vision they were working in”	Judgement: High Comment: No mention of blinding of assessors	Judgement: Low Comment: Not a significant amount of missing data. (*n* = 1)	Judgement: Low Comment: Report on all outcomes of interest	Judgement: High Comment: (1) May have been a disadvantage to control group “The probable reason for the unpleasantness of working with polarised glasses in 2D vision is that only one eye received an image through the polarised glasses, because only one of the two projectors emitted an image…This means that the students had binocular vision when working in 2D” (2) No mention of funding source or conflict of interest
Kikuchi et al. (2013)^28^	Judgement: Unclear Comment: Insufficient information about the sequence generation process. “The students were randomly assigned to one of three groups”	Judgement: Unclear Comment: No mention of how allocation occurred	Judgement: High Comment: Participants would likely be aware of using the intervention or not	Judgement: Low Comment: Data is unlikely to be affected by assessor's opinion; “The VRS was used to collect and analyse the data from the three groups. Consequently, all groups could be compared based on the same assessment”	Judgement: Low Comment: No missing outcome data reported	Judgement: Low Comment: Report on all outcomes of interest	Judgement: Unclear Comment: No mention of funding source or conflict of interest
Suebnukarn et al. (2012)^21^	Judgement: Unclear Comment: Insufficient information about the sequence generation process. “Students were then randomized to training on either the micro‐CT tooth models with a haptic VR simulator (*n* = 16) or extracted teeth in a phantom head (*n* = 16) training environments for 3 days, after which the assessment was repeated”	Judgement: Low Comment: “Participants were randomized using the closed envelope technique; that is, participants were given random allocations within closed opaque envelopes as to whether they performed their first endodontic microsurgery with or without virtual pre‐surgical practice (washout period = 1 month)”	Judgement: High Comment: Participants would likely be aware of using the intervention or not	Judgement: Low Comment: “The procedure itself was video‐recorded and blindly assessed by two experienced endodontists using an endodontic surgical competency rating scale modified from an objective structured assessment of technical skills (OSATS)”	Judgement: Low Comment: No missing outcome data reported	Judgement: Low Comment: Report on all outcomes of interest	Judgement: High Comment: (1) “A limitation of this study was the small sample size” (2) “Results are also limited by specificity to porcine teeth with no apical lesion” (3) No mention of conflict of interest
Suebnukarn et al. (2011)^25^	Judgement: Unclear Comment: Insufficient information about the sequence generation process. “Participants were randomized to experiment or control group”	Judgement: Low Comment: “A statistician not involved with the study undertook the randomization using sealed opaque envelopes ….”	Judgement: High Comment: Participants would likely be aware of using the intervention or not	Judgement: Low Comment: “The main outcome measure was procedural errors assessed by an expert blinded to trainee and training status”	Judgement: Low Comment: No missing outcome data reported	Judgement: Low Comment: Report on all outcomes of interest	Judgement: Unclear Comment: No mention of conflict of interest
Suebnukarn et al. (2010)^26^	Judgement: Unclear Comment: Insufficient information about the sequence generation process. “Stratified random sampling was used to allocate participants to the four conditions that received augmented kinematic feedback, such that each group had five females and three males…”	Judgement: Unclear Comment: No mention of how allocation occurred	Judgement: High Comment: Participants would likely be aware of using the intervention or not	Judgement: Low Comment: “All preparations were blindly evaluated and graded by the VR system, using six evaluation parameters (visibility of the canal orifices, four axial walls, and pulpal floor). The system assigned to each preparation an overall preparation score between 0 and 18”	Judgement: Low Comment: No missing outcome data reported	Judgement: Low Comment: Report on all outcomes of interest	Judgement: Unclear Comment: No mention of conflict of interest
